# Temporal genomic dynamics of sequence type 39 Klebsiella pneumoniae in a neonatal unit in Blantyre, Malawi

**DOI:** 10.1099/mgen.0.001673

**Published:** 2026-04-01

**Authors:** Allan M. Zuza, Oliver Pearse, Daryl B. Domman, Zoe A. Dyson, Kondwani Kawaza, Patrick Musicha, Nicholas A. Feasey, Eva Heinz

**Affiliations:** 1 Malawi Liverpool Wellcome Program, Kamuzu University of Health Sciences, Blantyre, Malawi; 2 School of Medicine, University of St Andrews, St Andrews, UK; 3 Department of Clinical Sciences, Liverpool School of Tropical Medicine, Liverpool, UK; 4 Biosciences Division, Los Alamos National Laboratory, Los Alamos, USA; 5 London School of Hygiene and Tropical Medicine, London, UK; 6 Wellcome Sanger Institute, Wellcome Genome Campus, Hinxton, UK; 7 Kamuzu University of Health Sciences, Blantyre, Malawi; 8 Strathclyde Institute of Pharmacy & Biomedical Sciences, University of Strathclyde, Glasgow, UK

**Keywords:** extended-spectrum beta-lactamase (ESBL), hospital-acquired infection (HAI), Sub-Saharan Africa

## Abstract

**Background***. Klebsiella pneumoniae* (*Kpn*) is an important cause of healthcare-associated infections (HAIs). In low- and middle-income countries, HAI due to *Kpn* disproportionately affects neonates. In this study, we investigated the genomic changes that occurred during long-term circulation of a *Kpn* sequence type (ST) 39 clone, causing a disproportionate number of infections on the neonatal ward at a tertiary healthcare facility in Malawi in 2017.

**Methods.** We analysed whole-genome sequences of *Kpn* ST39 collected from Queen Elizabeth Central Hospital over a 20-year period, including the generation of several high-quality hybrid genomes. We compared virulence markers, antibiotic resistance determinants and mobile genetic elements, focusing on variable regions between strains from the outbreak clone in 2017 and genomes from other co-occurring ST39 lineages.

**Results.** We identified eight variable genomic regions that demonstrate the plasticity of *Kpn* within ST, including the role of bacteriophages in shaping the genome of ST39.

**Conclusions.** The analysed *Kpn* ST39 lineages have a highly variable genome capable of incorporating large genomic regions during prolonged hospital circulation, which may offer a selective advantage in hospital environments and provide resistance to antimicrobial agents.

**Impact Statement**

*Klebsiella pneumoniae* is an important healthcare-associated pathogen, often circulating in hospital environments for prolonged stretches of time. Whilst a lot of studies describe outbreaks, we have limited insight into the changes that occur during an outbreak, including potential adaptation to the hospital or human host environment. This study is a detailed analysis of a *K. pneumoniae* lineage, part of sequence type (ST) 39, that caused a large number of neonatal sepsis cases in Queen Elizabeth Central Hospital (QECH), a tertiary healthcare facility in Blantyre, Malawi. We present an in-depth genome analysis of the clonal lineage and a comparison with other non-outbreak ST39 isolates from QECH, and highlight changes driven by mobile elements, including (pro)phages in the genome that indicate the acquisition of classic host-adaptation virulence factors such as adhesins.

## Data Summary

All new sequencing data are available in BioProject PRJEB102175; detailed accession numbers are provided in Table S2; and the accessions used for the already published short-read data are present in Table S1. The authors confirm that all supporting data, code and protocols have been provided within the article or through supplementary data files.

## Introduction

*Klebsiella pneumoniae* (*Kpn*) is a significant cause of healthcare-associated infections (HAIs) globally [1,2]. HAIs due to *Kpn* include lower respiratory, urinary tract and bloodstream infections, as well as meningitis and surgical site infections [1,3, 4]. *Kpn* infections are increasingly resistant to the World Health Organisation (WHO)-recommended antibiotic regimes, and carbapenem-resistant *Enterobacteriales*, including *Kpn*, are designated ‘critical priority’ by the WHO [5,6]. Antibiotic resistance in *Kpn* is driven by mobile genetic elements such as transposons, bacteriophages and conjugative plasmids, as well as, less frequently, chromosomal mutations. Mobile elements can carry genes for resistance to multiple drug classes, rapidly leading to multidrug-resistant lineages [2]. Mobile elements are also important for the spread of virulence determinants, which are important for host invasion, leading to clones that can cause life-threatening infections in human hosts [2]. Some of the well-described virulence factors are the siderophores salmochelin, aerobactin, yersiniabactin and enterobactin, which are linked to *Kpn* invasive disease [7].

In low- and middle-income countries, *Kpn* has emerged as a major cause of neonatal infection and a frequent cause of outbreaks. Large multinational studies in Africa and East Asia show that *Kpn* is the number one cause of neonatal sepsis and also a leading cause of childhood deaths [8,9]. In Malawi, more than half of all *Kpn* invasive disease over a 20-year period at Queen Elizabeth Central Hospital (QECH) in Blantyre, Malawi, occurred in neonates [10]. During this time, *Kpn* lineages have undergone continuous replacement over the years, with sequence type (ST) 15, ST14 and ST39 comprising the majority of *Kpn* invasive disease at QECH [10], which have been observed to cause outbreaks in neonatal and paediatric wards [11]. However, we do not fully understand whether genomic changes take place and whether these contribute to the success of specific STs in a ward environment where multiple STs are circulating [10,11].

In this study, we investigated whether a clonal *Kpn* ST39 lineage caused the majority of all invasive disease in neonates at QECH in 2017 and identified regions of interest that are candidates for further investigation of success within the hospital environment, interpreted as causing a majority of infections. We performed a high-resolution genomic analysis, including the generation of several high-quality reference genomes, to explore the circulating ST39 genome plasticity at full resolution, including integration sites of mobile genetic elements, which in most cases cannot be confidently resolved with short-read-only data.

## Methods

### Samples analysed in this study

This project describes a detailed analysis of a subset of all *Kpn* isolates from blood and cerebrospinal fluid (CSF) samples collected from patients seeking routine care at QECH between 1998 and 2020, and stored in the Malawi Liverpool Wellcome (MLW) Programme biobank. Routine (ISO15189-accredited since 2019) diagnostic blood culture services have been provided to the medical and paediatric wards by the MLW Programme since 2000. For adults, 7–10 ml of blood was collected from all patients admitted to the hospital with fever (axillary temperature >37.5 °C) or with clinical suspicion of sepsis, severe sepsis or septic shock. Sepsis, severe sepsis or septic shock was suspected in patients with tachycardia (≥90 beats per minute), hypotension (systolic blood pressure <90 mm Hg), tachypnoea (respiratory rate >20 min^−1^) or delirium. Three to ten millilitres of blood was taken from children with a non-focal febrile illness who tested negative for malaria, who were severely ill with suspected sepsis or who failed initial malaria treatment and remained febrile. For adults and children with clinical suspicion of meningitis (temperature >37.5 °C with seizures, headache, abnormal behaviour or meningism), a lumbar puncture was also performed, and the sample was sent for CSF analysis. For neonates with clinical signs of sepsis or meningitis (temperature >37.5 °C or other signs of clinical deterioration such as reduced activity, increasing work of breathing or respiratory rate >60, heart rate >180 or <100 and unexplained seizures), a blood culture and CSF sample were collected. Afebrile patients were unlikely to have had blood or CSF cultures unless the clinical suspicion of sepsis or meningitis was high. For blood cultures, samples were collected using aseptic methods and inoculated into a single aerobic bottle (BacT/ALERT; bioMérieux, Marcy-L’Etoile, France). These were incubated using the automated BacT/ALERT system (bioMérieux, France) since 2000, before which they were cultured manually. Samples that flagged positive were Gram-stained, and Gram-negative bacilli were identified using the Analytical Profile Index (bioMérieux). CSF samples were processed for cell count and biochemistry before manual culture. The *Kpn* genomes analysed in this study were focused on ST39 isolates, given that ST39 caused 53.2% of all invasive disease *Kpn* cases at QECH in 2017 [10]. QECH is one of the two tertiary hospitals in southern Malawi offering free-of-charge medical care to Blantyre and referrals from the surrounding districts. The ST39 genomes analysed in this study were mainly isolated from Chatinkha nursery, which is the neonatal unit at QECH. All wards at QECH have access to blood and CSF culture services offered by the MLW, which has provided diagnostic services to QECH since 1998. We retrieved the Illumina short reads of these genomes from the European Nucleotide Archive (ENA) (Table S1, available in the online Supplementary Material) [10].

### Oxford Nanopore sequencing

We selected five representative isolates, two (BKREGE, CAA8N9) belonging to the outbreak lineage and three (1004078, CAAUE8, CHI11E) belonging to the lineages that only caused sporadic infections, to use long-read sequencing on the Oxford Nanopore Technologies MinION platform for complete assemblies; the long-read sequences of CHI11E had already been published as part of the long-term study [10]. We extracted DNA using the MasterPure Complete DNA and RNA Purification Kit (Biosearch Technologies, UK) and performed quality control of the extracted DNA using the Qubit 4.0 fluorometer (Thermo Fisher Scientific Inc.). We prepared the sequencing library using the SQK-RBK004 rapid library preparation kit (Oxford Nanopore Technologies plc) and sequenced on the MinION Mk1c device using the R9.4.1 flow cell. We acquired data from the sequencer using MinKNOW^TM^ software (Oxford Nanopore Technologies plc) and used the Guppy basecalling software (version 6.0.7+c7819 bc; Oxford Nanopore Technologies plc) for basecalling and demultiplexing. We ran Guppy with the default settings using the super-accurate (sup) basecaller mode.

### Quality control and assembly

For the reads retrieved as above, and from SRR28748993 (CHI11E), we used NanoPlot (v1.38.1) [12] and FastQC (v0.12.1) [13] to perform ONT (Oxford Nanopore Technologies) and Illumina read quality assessments, respectively. We trimmed and filtered the ONT reads using Chopper (v0.9.0) with the ‘-q 10 l 500’ flags to keep reads longer than 500 nucleotide bases and with at least a Phred quality score of 10 [12]. The quality metrics of the reads used in the analysis are available in Fig. S1. We performed *de novo* assemblies of samples with only Illumina reads using SPAdes (v3.15.5) [14,15], implemented in Shovill (v1.1.0) [16]. We generated hybrid assemblies for the five strains for which both short-read Illumina data and long-read ONT data were available, using the Trycycler (v0.5.4) assembly pipeline [17]. The pipeline generated the initial assemblies using Flye (v2.9.1-b1780) [18], Raven (v1.5.0) [19] and MiniPolish (v0.1.2) [20]. We polished the consensus genome using Medaka (v1.11.3) [21], Polypolish (v0.5.0) [22] and POLCA (MaSuRCA v4.1.0) [23]. We annotated all the assemblies using PROKKA (v1.14.6) with the ‘--usegenus --genus *Klebsiella* --addgenes --force –compliant’ parameters [24]. All accessions of the hybrid assemblies and their underlying reads are provided in Table S2; quality control determinants of long- and short-reads are given in Fig. S1A and B, respectively.

### Bacterial genome typing

To identify antimicrobial resistance genes (ARGs) and plasmid replicons, we used AMRFinder Plus (v4.0.15, Database version 2024-12-18.1) [25] with the ‘–plus’ flag and Abricate (v1.0.1) [26] with the PlasmidFinder database (Database date: 4 November 2023) [27] to detect plasmid replicons in the assemblies, respectively. We reconstructed plasmids from Illumina assemblies using mob_recon (v3.1.9) [28]. We identified prophage regions using PHASTER [29] and explored the identified regions using PhageScope [30]. We used Kleborate (v3.1.3) to identify *Kpn*-specific virulence factors, capsule (K-) and lipopolysaccharide O-antigen (O-) types [31].

### Phylogenetic analysis

We performed a whole-genome core SNP analysis using Snippy (v4.6.0) with the outbreak genome BKREGE as the reference [32]. We created a multiple sequence alignment of all genomes using Snippy. We identified and masked recombinant regions using Gubbins (v3.4) [33]. We constructed a maximum-likelihood phylogenetic tree from the recombination-free alignment with 1,552 sites using IQTree (v2.2.2.7) [34,35]. We ran IQTree with 1,000 bootstrap replicates and the General Time-Reversible plus gamma (GTR+G) nucleotide substitution model using the recombinant-free alignment generated by Gubbins. To define sub-lineages from the constructed tree, we used rPinecone (v0.1.0) [36]. We rooted the tree at midpoint using the phangorn (v2.11.1) [37] package in R (v4.2.1, R Development Core Team, 2022) [38] and visualized it using the ggtree R package [39].

To estimate the time of divergence for strains in the outbreak clade, we constructed a time-dated phylogenetic tree using the Bayesian Evolutionary Analysis Sampling Trees (BEAST, v1.10.4) software [40]. We checked the alignment for a phylogenetic signal using TempEst (v1.5.3), as shown in Fig. S2 [41]. BEAST was run with the GTR+G model with the empirical site heterogeneity rates, the relaxed clock model and the coalescent constant-sample-size option. A chain length of 800,000,000 was selected for the analysis, with logging every 5,000 states. We ran BEAST in three replicates and combined their outputs using LogCombiner (v1.10.4) to generate a maximum clade credibility tree [40].

### Statistical testing and plotting

All statistical testing was performed in the R Statistical Computing software (v4.2.1). To determine whether the cases of ST39 observed in 2017 were higher than expected, we modelled the yearly counts of ST39 cases using a negative binomial model. We estimated the probability of observing the number of cases in 2017 by fitting the 2017 cases to the mean and variance of the modelled yearly counts using the 'pnbinom' function from the 'stats' R package [38]. We used the ggpubr (v0.6.0) package to test for differences in the distribution of plasmid replicons and ARGs between the different clades [42]. Data wrangling and plotting were generated using the tidyverse packages (v1.3.2) [43], ggpubr, ggblanket (v12.2.0) [44] and patchwork (v1.2.0) [45].

## Results

### Outbreak in Chatinkha nursery

**Fig. 1.**
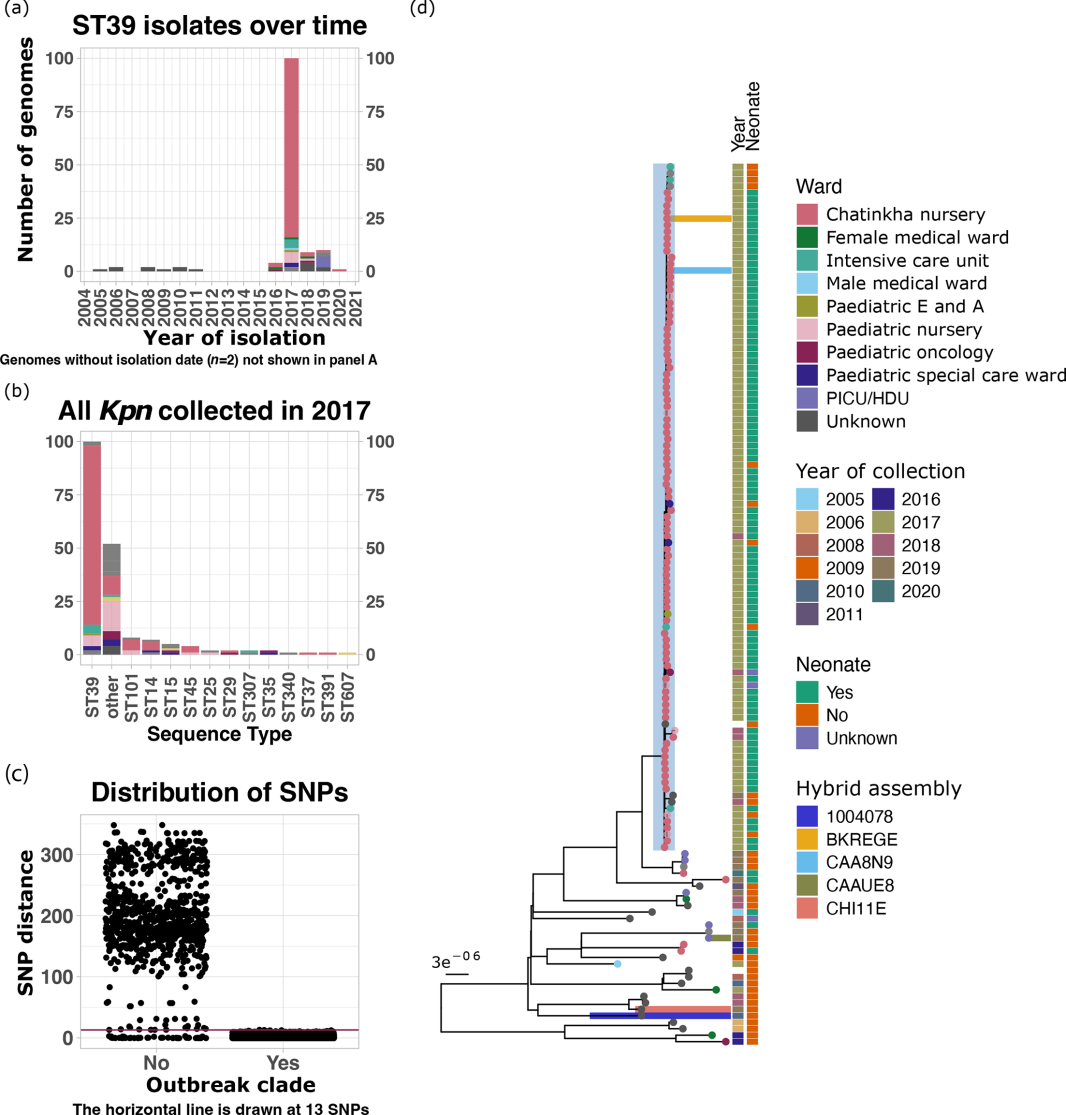
(a) Distribution of all ST39 genomes analysed in this study across the years; the ward of sample collection is shown by the colour of the bars. (b) Distribution of all *Kpn* isolates collected in 2017 reported previously, highlighting the contrast in the number of ST39 isolates vs other STs. The bars are coloured by the ward of sample collection. (c) Distribution of pairwise SNP distances within the outbreak and non-outbreak genomes. (d) A maximum-likelihood phylogenetic tree constructed from whole-genome core SNPs. The tips are coloured by the ward of sample collection, and the year of sample collection and patient groups are mapped to the right of the phylogenetic tree. The isolates for which we obtained long-read sequence data are highlighted by horizontal bars from the tips of those genomes. The shaded branch represents the outbreak clade. HDU, high-dependence unit; Paediatric E and A, Paediatric Emergency and Accident; PICU, paediatric intensive care unit.

In a large longitudinal study analysing the collection of invasive *Kpn* isolates from QECH, we observed a marked increase in *Kpn* ST39 cases in 2017 (*n*=100/188; *P*=3.157208 e^−11^). The number of cases later reduced to 9/208 in 2018, and 10/236 cases were observed in 2019 (Fig. 1a) [10]. This observed increase in cases in 2017 led us to investigate the genomes of the ST39 lineage.

We analysed 135 *Kpn* ST39 genomes, which were collected between 2005 and 2020 (Fig. 1a). These genomes were collected from nine wards: one neonatal (Chatinkha nursery), five paediatric wards (Paediatric Emergency and Accident, paediatric nursery, paediatric oncology, paediatric special care ward and paediatric intensive care unit/high-dependence unit) and three adult wards (female medical ward, intensive care unit and male medical ward). The majority of the ST39 genomes were isolated in 2017 (74.1%, *n*=100). The ST39 genomes from 2017 were primarily isolated from Chatinkha nursery (84%, *n*=84). This proportion contrasts with other STs, where Chatinkha nursery accounted for only 27.3% of cases of all other genomes from 2017. This was not due to differences in the number of samples collected from Chatinkha nursery in 2017, as all other STs remained in very low numbers in 2017 (overall <8 cases per ST and <5 cases in Chatinkha nursery) (Fig. 1b).

Phylogenetic analysis revealed that 98% (*n*=98) of the genomes from 2017, 5 genomes from 2018, 1 genome from 2019 and another genome with a missing date of collection formed a monophyletic branch with less than 13 SNPs differentiating them [median pairwise SNP distance of 2 (0–13)] (Fig. 1c and d, Fig. S3). Genomes on this branch are therefore referred to as the outbreak clade genomes throughout this paper. We also observed that the genomes within the outbreak clade that were collected in 2017 all belonged to neonatal and infant wards (Fig. S4). Only two genomes from 2017 were not included in the outbreak clade, and these were collected from adult wards.

### ARGs and virulence determinants in outbreak genomes vs non-outbreak genomes

**Fig. 2.**
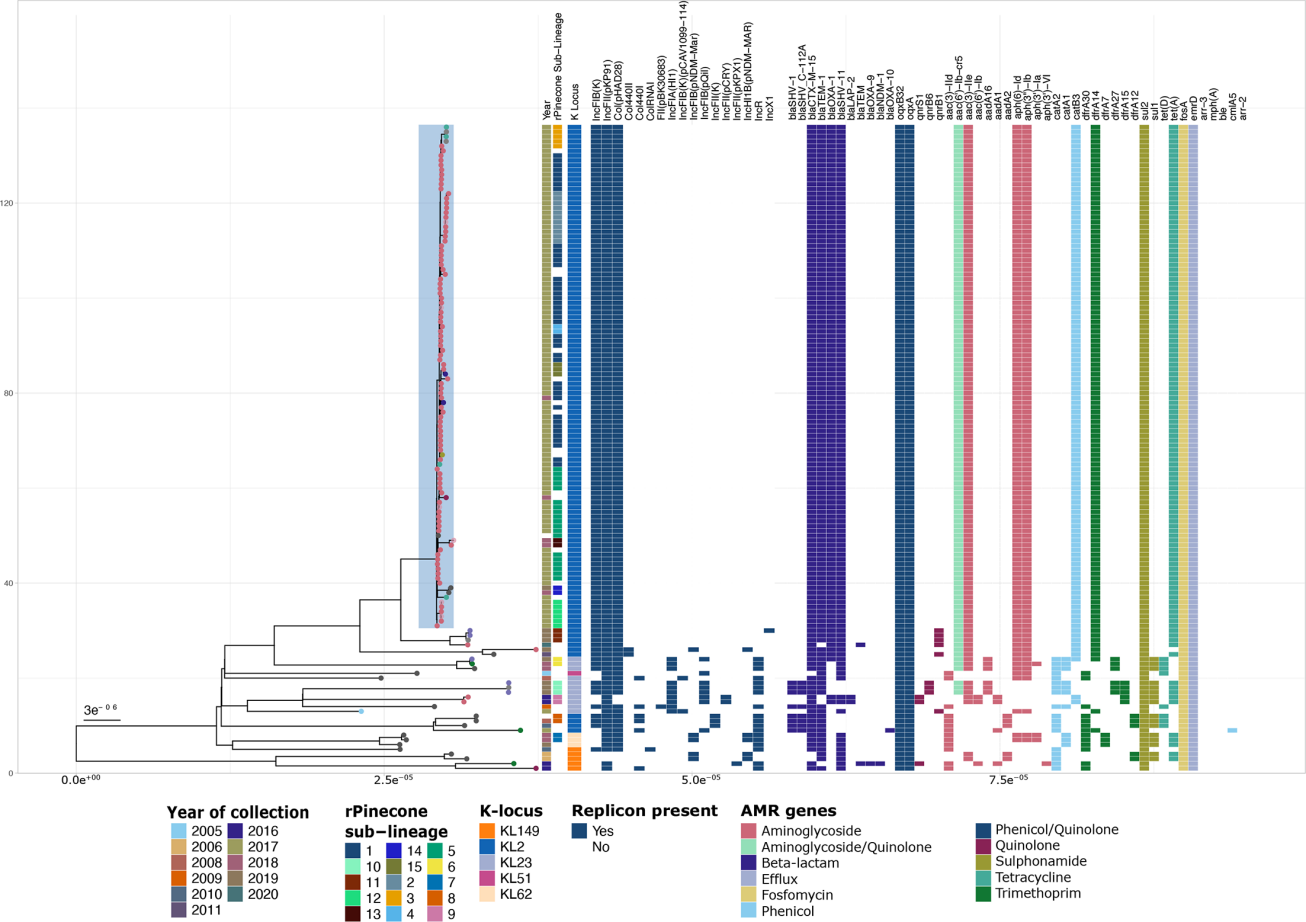
A maximum-likelihood phylogenetic tree of all genomes in the study showing the year of isolation, rPinecone sub-lineage, K-locus type, presence of plasmid replicons and ARGs, displayed as a heatmap mapped to the right of the phylogenetic tree.

**Fig. 3.**
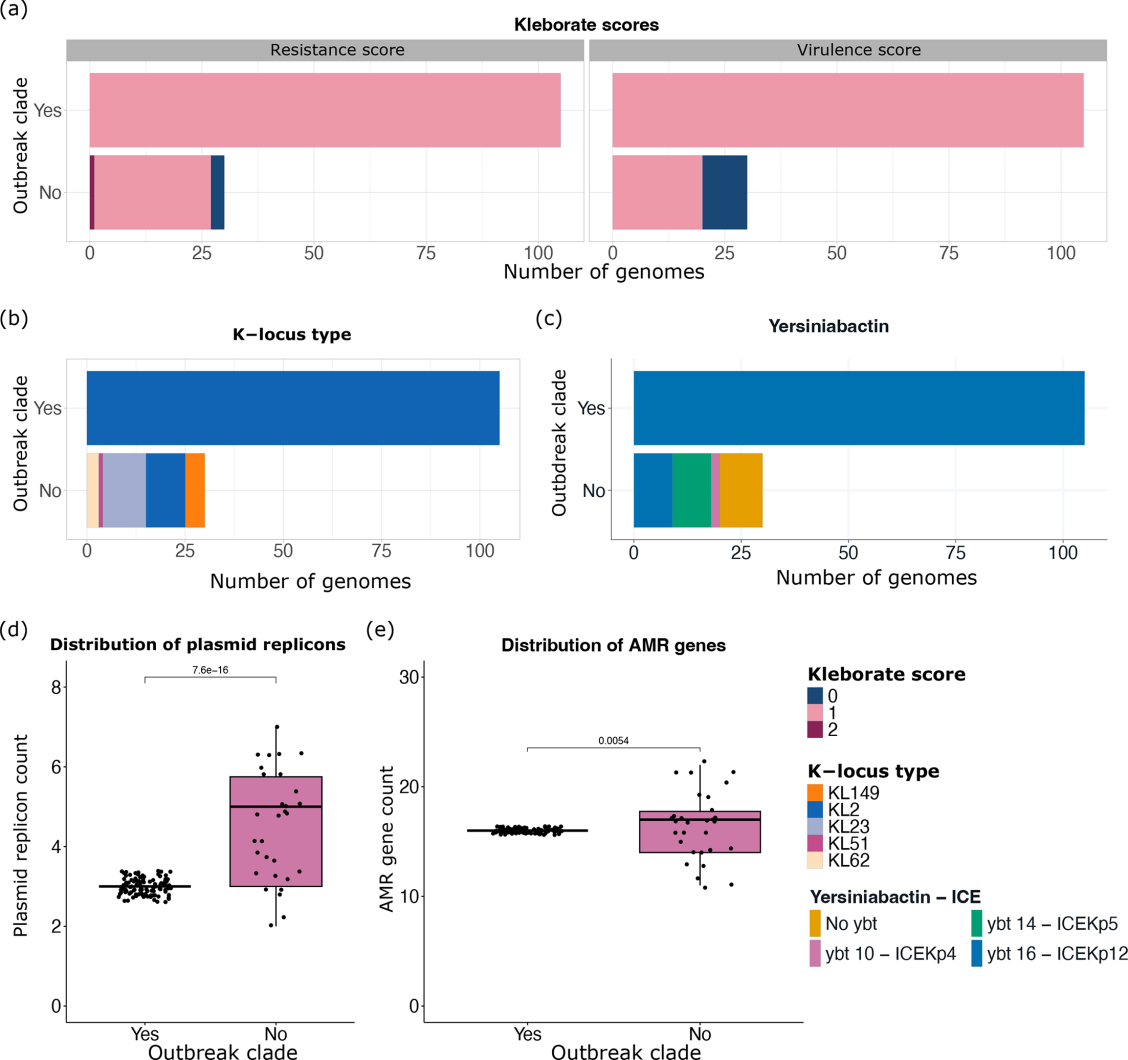
Comparison of the distribution of the number of AMR genes. (a) Distribution of virulence and resistance scores calculated by Kleborate. A resistance score of 1 represents the presence of ESBL, and 2 represents the presence of a carbapenemase. Genomes that do not encode genes for ESBLs, carbapenems and colistin are assigned a resistance score of 0 by Kleborate. The virulence score of 1 represents the presence of one virulence operon; in our study, this represents yersiniabactin only, and 2 represents yersiniabactin and colibactin in our collection. Genomes that lack the siderophores yersiniabactin, colibactin, aerobactin and salmochelin, as well as the hypermucoid loci rmpA, are assigned a virulence score of 0. (b) Distribution of K-locus types. (c) Distribution of yersiniabactin ST. (d) Distribution of plasmid replicons per genome. (e) Distribution of ARGs identified per genome between the outbreak clade genomes and non-outbreak clade genomes. ESBL, extended-spectrum beta-lactamase; ICE, integrative and conjugative element*.*

Overall, genomes in this study encoded a total of 47 distinct ARGs (Fig. 2), and we did not identify changes in the predicted resistance or virulence potential in the outbreak clade compared with others (Fig. 3a), but we observed less variation in capsule or virulence types, as expected in a clonal outbreak (Fig. 3b and c). All genomes in this study encoded the O1ab O-type. We noted variability in the K-types, with all isolates in the outbreak clade being K2 (KL2 locus) and other isolates having one of the following K-types: K23 (KL23), K52 (KL51), K62 (KL62) or unknown (KL149) (Fig. 3b).

In detail, we identified ARGs to three subclasses of beta-lactam antibiotics: beta-lactams (*bla_SHV-1_*, *bla_TEM-1_*, *bla_SHV-11_*, *bla_LAP-2_*, *bla_TEM_* and *bla_OXA-9_*), cephalosporins (*bla_SHV_C-112A_*, *bla_CTX-M-15_*, *bla_OXA-1_* and *bla_OXA-10_*) and carbapenems (*bla_NDM-1_*) (Fig. 2). Other classes of ARGs identified in these genomes include aminoglycoside [*aac (3)-IId*, *aac (3)-IIe*, *aph (6)-Id*, *aph(3'')-Ib*, *aph(3')-Ia*, *aadA16*, *aadA1*, *aac(6')-Ib*, *aadA2*, *aph(3')-VI* and *aac(6')-Ib-cr5*], fluoroquinolone (*qnrS1, qnrB6, qnrB1*), fosfomycin (*fosA*), macrolide [*mph(A*)], chloramphenicol (*catA1*, *catA2*, *catB3*, *cmlA5*), rifamycin (*arr-2*, *arr-3*), sulphonamide (*sul1*, *sul2*), tetracycline [*tet(A)*, *tet(D*)] and trimethoprim resistance genes (*dfrA7*, *dfrA12*, *dfrA14*, *dfrA15*, *dfrA27*, *dfrA30*) (Fig. 2).

The carbapenemase *bla_NDM-1_* was carried by a single genome from the non-outbreak clade (ERR12058819) (Fig. 2). This sample had six plasmids reconstructed by mob_recon, two mobilizable and four non-mobilizable. The *bla_NDM-1_* gene was located on a 98 kb IncFII(pKPX1) conjugative plasmid. The plasmid was most similar to the published plasmid sequence CP023910.1 (100% nucleotide identity, 100% query coverage) [46]. This plasmid encoded 47.3% (9/19) of the ARGs identified in this genome [*bla_NDM-1_*, *aac(6')-Ib*, *aadA1*, *aph(3')-VI*, *bla_CTX-M-15_*, *blaOXA-9*, *bla_TEM_*, *ble*, *qnrS1*].

We observed a total of 17 plasmid replicon types in the collection. The genomes in the outbreak clade had a uniform composition of three replicon types, including IncFIB(K), IncFII(pKP91) and Col(pHAD28). The non-outbreak genomes had varying plasmid replicon types and numbers, with a median of 5 (2–7) plasmid replicon types per genome (Fig. 3d). Similarly to ARGs, the number of plasmid replicons was also lower in the outbreak clade genomes (Fig. 3d and e), although this did not impact the number of antimicrobial classes these isolates are predicted to be resistant against (Fig. 2).

### Temporal analysis using BEAST

**Fig. 4.**
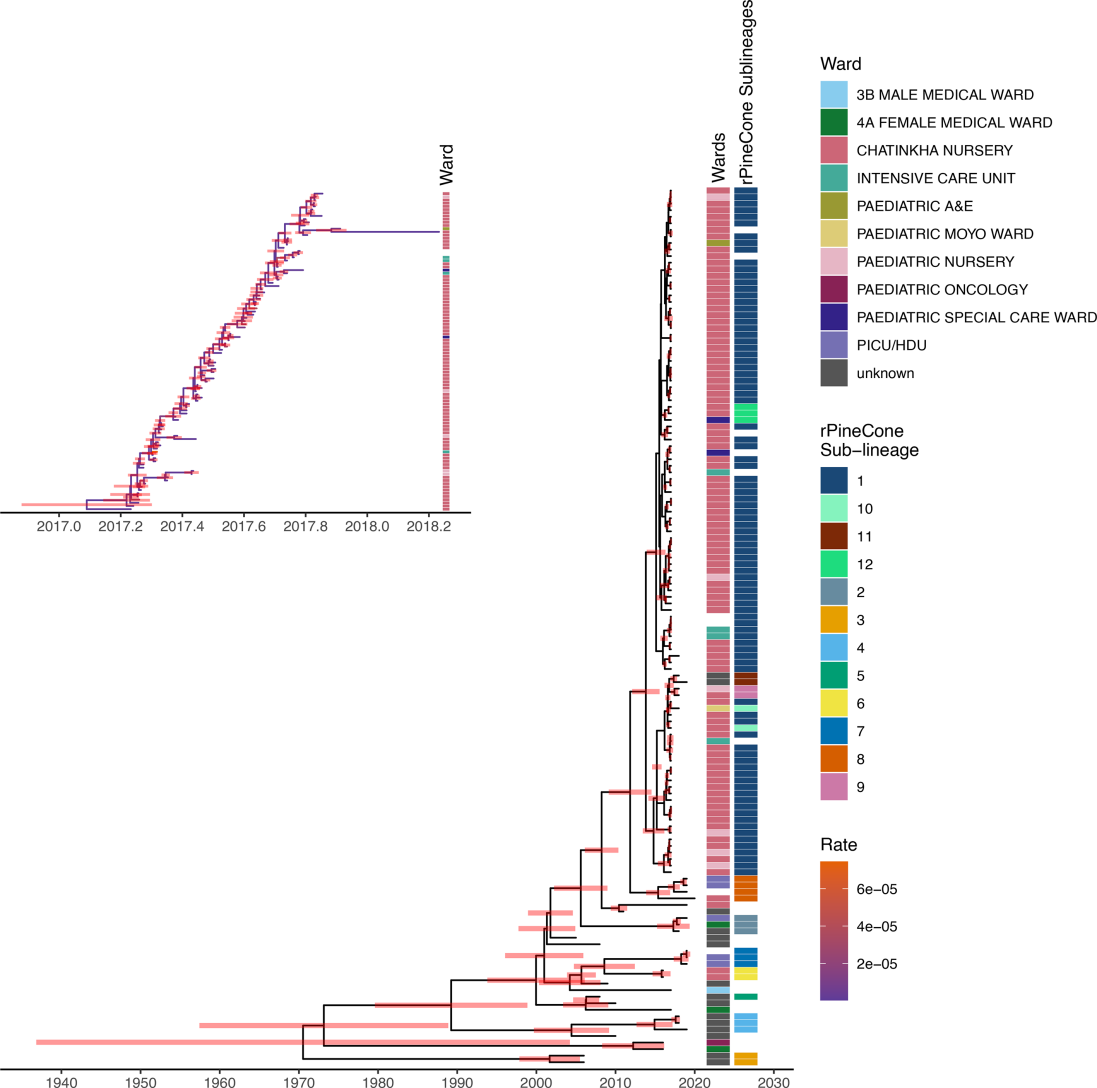
A time-dated phylogeny showing the time of divergence of the outbreak clade. The highest posterior densities are shown as bars on the nodes. The ward of isolation and rPinecone sub-lineages are mapped to the right of the tree. The inlet shows genomes from the outbreak clade that had complete isolation-date information; six genomes from the outbreak clade were missing the date of isolation. The inlet shows the ward of sample collection mapped to the right. The branches are coloured by branch rates. HDU, high-dependence unit; Paediatric E and A, Paediatric Emergency and Accident; PICU, paediatric intensive care unit.

We observed that the outbreak clade shared the most recent common ancestor (MRCA) with another clade that continued to circulate in the hospital after 2017 (Fig. 4). The MRCA for these two clades was in November 2013 (95% highest posterior density interval of March 2012 to August 2015). This divergence occurred 4 years before the outbreak clade genomes were isolated at QECH. The strains in the outbreak clade consist of two groups of genomes: 2017 outbreak genomes, which were isolated each month between March 2017 and November 2017, and sporadic cases in 2018 (one in January, two in March and one in October) (Figs 4 and S4). The clade that shared the MRCA with the outbreak clade continued to cause infections in the neonatal wards after the outbreak. Genomes in this adjacent clade are available in our data up to April 2020. All cases in this clade clustered together phylogenetically and formed an rPinecone sub-lineage 8 (Fig. 4).

### Genomic plasticity observed between outbreak and sporadic isolates

**Fig. 5.**
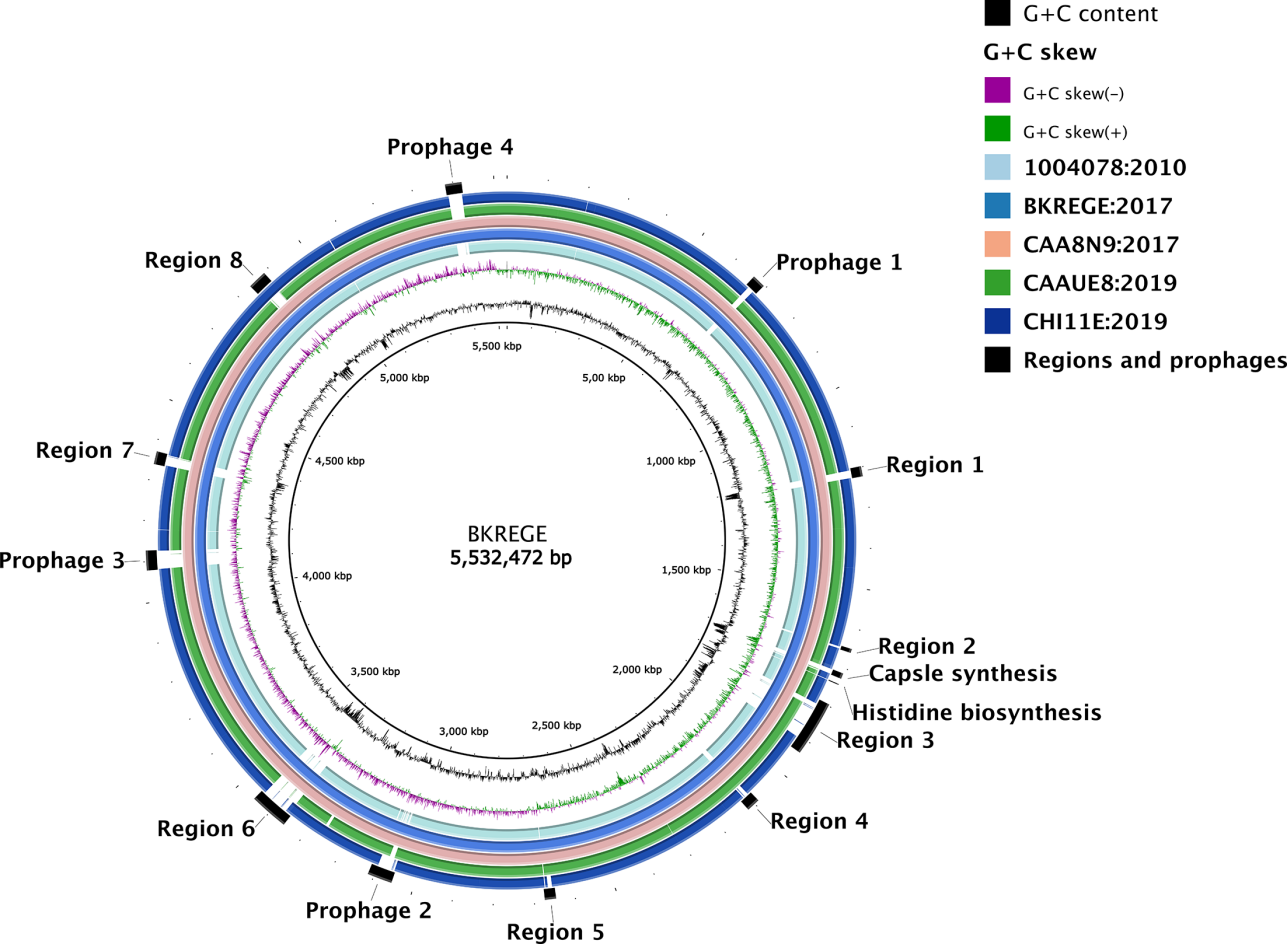
A gene map showing a comparison of chromosomes for the hybrid assemblies, highlighting prophage sequences and variable regions when the assemblies are mapped to the reference genome, BKREGE. Each ring represents a chromosome in the same order as the legend.

We identified a total of 13 variable regions when the hybrid assemblies are compared, using BKREGE, one of the two closed outbreak genomes, as the reference strain (Fig. 5). Four of these regions contained prophage sequences, one was the capsule biosynthesis region, and the remaining included regions coding for a variety of products. Six regions (prophage 1 and 3, and variable regions 3, 6, 7 and 8) were located next to tRNA loci (Fig. 5).

Focusing on the variable regions that did not include prophage sequences, we noted a size range from 9 to 138 kb (Fig. S5). While most of these contained coding sequences (CDSs) for proteins of unknown function (hypothetical proteins), three regions (variable regions 3, 6 and 8) contained CDSs with known function (Fig. S5). These three variable regions were present in all outbreak clade genomes but only in a subset of non-outbreak genomes at varying degrees (Fig. S6). Variable region 3 (Figs 5 and S6) is a 120 kb region and includes a type IV secretion system, CDSs involved in iron import (*irtA*), vitamin B12 import (*btuD*) and a manganese transport operon (*mntB*, *mntH*, *mntA*, *mntC, mntP*, *mntR*, *mntS*, *scaC*). Variable region 6 (Figs 5 and S6) is an 86 kb region with a toxin/anti-toxin system (*cbtA*, *cbtE*), antigen 43 (*flu*) that controls autoaggregation in *Escherichia coli*, an antirestriction protein (klcA), capsular F1 antigen usher/chaperon (*caf1A and caf1M*) first isolated in *Yersinia pestis* to assist in cell surface adhesion, vitamin B12 import (*btuD*) and ferric erobactin receptor (*fepA*) [47,48]. The variable region 8 (Fis 5 and S6) is 46 kb and mainly contains CDSs of unknown function. This region includes a toxin–antitoxin system (*ykfI, yfjZ*), an anti-restriction protein (*klcA*) and a type I restriction enzyme protein (*hsdS*).

Given the differences in prophages observed (Fig. 5), we then assessed each hybrid assembly individually for prophage sequences and identified nine genomic regions with prophages predicted in some or all of the strains (Figs S7 and S8). The integrated regions ranged in size from 6 to 56 kb, with the smaller ones being from the *Inoviridae* family of phages (*n*=2, 6 and 9 kb) (Figs 5 and S7).

Two large prophage regions are inserted only in the two outbreak isolates (BKREGE and CAA8N9); these regions encode only hypothetical proteins, and their impact on the strains is yet to be determined (Supplementary Fig. S8A, B). Two further large regions are similar between the outbreak isolates and the non-outbreak isolate CAAUE8 (Figs 1d, S8C and D); one of these (Fig. S8D) encodes several virulence and heavy-metal resistance genes.

Two predicted prophage regions are somewhat extended in the outbreak isolates compared with some of the other genomes (Fig. S8E,F), but contain more genes in one of the non-outbreak isolates, 1004878 (Fig. S8E) and CHI11E (Fig. S8F), respectively. Finally, a prophage region slightly expanded in the outbreak isolates also includes the *oqxAB* operon (Fig. S8G), which encodes a multidrug efflux pump [49]. This region is known to be conserved in *Kpn* as species, and prophage predictions do not always include the *oqxAB* operon due to differences in the Att site recognition up- or downstream of the operon, leading to its inclusion or exclusion from predicted prophage regions, respectively.

**Fig. 6.**
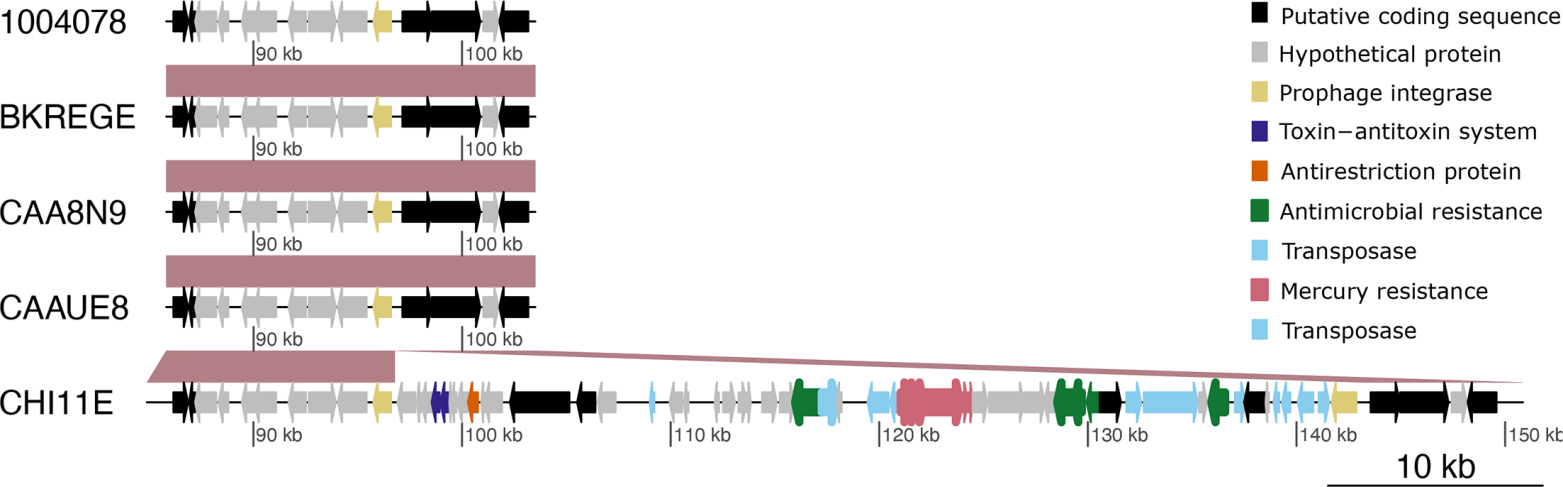
Chromosomal integration of ARGs and mercury resistance genes. The arrows represent CDSs.

Two further predicted prophages are present only in one of the non-outbreak isolates each. Whilst the first (Fig. S8H), present in 1004878, is composed exclusively of hypothetical genes, the second region (Fig. S8I) in CHI11E carries a ~50 kb ARG island (Figs 6 and S9). The ARG island is inserted between a prophage integrase (*intS*) and a *tRNA-Sec* RNA at position 96757 of the reference strain BKREGE and includes ARGs *tet(A)*, *sul1*, *dfrA7* and *catA1*, as well as a mercury resistance operon (Fig. 6). The ARG island in CHI11E was seen in two other non-outbreak genomes isolated in 2018, which clustered together into a single clade, separated by a small genetic distance (<22 SNPs) (Fig. S9).

## Discussion

Here, we performed a detailed genomic analysis of ST39 *Kpn* genomes, which caused an outbreak in a neonatal unit in Blantyre, Malawi. We utilized a combination of short- and long-read sequences to perform a detailed interrogation of the genomes in the outbreak. Using a reference genome assembly from the outbreak isolates allowed us to obtain a high level of resolution to identify the genomic changes in this lineage. The utility of hybrid assemblies also allowed us to identify chromosomal integration of ARGs when comparing outbreak with non-outbreak isolates, highlighting the plasticity of antimicrobial resistance (AMR) determinants and their impact on chromosomal structure. Our data suggest that chromosomal insertions are not randomly distributed but are concentrated in certain locations along the genome of the ST39 lineage.

Our data also show the plasticity of prophage regions within this ST, and that these regions remain important chromosomal locations for integration of future bacteriophages, as highlighted by the presence of multiple prophage integrases in certain regions of the hybrid assemblies [50]. These regions are also important for AMR and virulence determinants, as prophages can integrate cassettes carrying cargo genes, which can persist and transmit vertically, as indicated by our data [51]. The plasticity highlights these as important genomic locations providing opportunities for acquisition of genetic material, including ones that can contribute to niche adaptation, which we speculate could be the case in our study, given the acquisition of heavy-metal metabolism loci (often linked to AMR) and ARGs.

The large number of insertion sequences, prophages and secretion systems identified in the variable regions suggests that these regions were acquired through mobile genetic element integration (and loss) in the genome of ST39. The presence of genes encoding antigen 43 and fimbriae-usher encoding genes in variable region 6 of the outbreak clade is of high interest, as these genes are known to be of relevance for adhesion to host cells and biofilm formation in *Enterobacteriaceae* [46]. Acquiring additional adhesion systems could allow the clone to have a competitive advantage in the human host over other circulating strains of *Kpn* or other opportunistic pathogens.

The *bla_NDM-1_* carbapenemase identified in an isolate from this collection highlights the risk of stable introduction of carbapenem-resistant *Kpn* into Malawi, against which only very limited treatment options are available [47]. The presence of distinct plasmids carrying carbapenemases in *Enterobacteriaceae* from Malawi indicates independent introductions into the country [48]. While carbapenemases have been reported in a few studies from Malawi [10,48, 52], clinical data show that meropenem usage in hospitals is common [53,54]. A study that analysed hospital records from four tertiary Malawian hospitals (including QECH) showed that *Kpn* resistance rates were as high as 7.4% [53]. This discrepancy between the phenotypic and genomic data highlights the need to expand genomic surveillance to other parts of Malawi for a more complete picture of AMR. It is also noteworthy that the data are largely reliant on public hospitals and linked pharmacies, whilst use in private hospitals and private for-profit wholesalers/distributors could not be captured in the national report [54].

Using these ST39 genomes as an example of a lineage that expanded at QECH, leading to an outbreak [10], highlights the variability within closely related *Kpn* isolates that would not be detected by core genome SNP analyses alone. A recent study from the same location observed ST39 *Kpn* in a wide range of samples, including the ward environment, indicating the ability of this lineage to persist in the environment and cause infections, as ST39 and other *Kpn* STs were observed circulating in the ward environment within 28 days of either invasive disease or stool colonization episodes [55]. We note that descendants from our outbreak lineage are still present in the ward environment in this later study, though isolates from other lineages dominate (Fig. S10); it is, however, challenging to draw conclusions given the temporal gap between our isolates and the environmental samples, and the lack of environmental sampling during 2017 (Fig. S11).

Our analysis also showed that the clone implicated in the outbreak may have diverged 3–8 years from its neighbouring clades before being introduced into and isolated at QECH, suggesting possible community circulation of this lineage before it was transferred to the hospital, or introduction of this ST39 clade from other parts of the world. Given the clonality of the outbreak, a single source or carrier, consistent with this, could potentially explain why the outbreak was interrupted somewhere in the first quarter of 2018, possibly due to the removal of the environmental reservoir. Although we were unable to ascertain the source of the outbreak in the hospital, it has been reported in our setting that *Kpn* frequently transmits between babies and mothers/guardians [55,56]. The hospital environment in the neonatal wards has also been known to be a risk factor for extended-spectrum beta-lactamase colonization [55]. It is therefore important to disrupt the movement of *Kpn* and other pathogens through access to and implementation of appropriate infection prevention and control (IPC) measures.

A limitation is the lack of clinical data to compare outcomes of infection between isolates from the outbreak clade and isolates from other ST39 lineages. The genomes are not linked to unique patient identifiers, and some patients may have multiple isolates, leading to an overestimation of the number of patients affected by the outbreak. Our data end at the beginning of 2020, and we were unable to determine if the AMR insertion into the chromosome persisted in the lineage, which will be of high interest for follow-up studies to explore the fixation of *Kpn* ST39 as a multidrug resistant(MDR) clone.

Further work, including phenotypic studies, on strains that undergo expansion in hospital environments to understand the impact of acquired genomic material on the phenotype of the strains will be of high interest. Meanwhile, better IPC measures need to be put in place to shield vulnerable populations, including neonates, from getting exposed to *Enterobacterales* circulating in hospital environments. We also need close to real-time genomic surveillance to identify outbreaks early and put in place extra control measures to limit the spread of outbreak clones and protect susceptible individuals.

## Supplementary material

10.1099/mgen.0.001673 Uncited Supplementary Material 1.

10.1099/mgen.0.001673 Uncited Supplementary Material 2.

## Abbreviations

AMR: antimicrobial resistance
ARGs: antimicrobial resistance genes
BEAST: Bayesian Evolutionary Analysis Sampling Trees
CDSs: coding sequences
CSF: cerebrospinal fluid
ENA: European Nucleotide Archive
ESBL: extended-spectrum beta-lactamase
GTR+G: General Time-Reversible plus gamma
HAIs: healthcare-associated infections
HDU: high-dependence unit
ICE: integrative and conjugative element
*Kpn*: *Klebsiella pneumoniae*
MLW: Malawi Liverpool Wellcome
MRCA: most recent common ancestor
Paediatric E and A: Paediatric Emergency and Accident
PICU: paediatric intensive care unit
QECH: Queen Elizabeth Central Hospital
ST: sequence type
WHO: World Health Organisation
